# Traumatic Superficial Femoral Arteriovenous Fistula with Pulsatile Mass and Leg Pain 60 Years after Stabbing Injury

**DOI:** 10.3400/avd.cr.22-00033

**Published:** 2022-06-25

**Authors:** Kazuki Takahashi, Shinsuke Kikuchi, Ai Tochikubo-Suzuki, Yuri Yoshida, Daiki Uchida, Atsuhiro Koya, Kazuya Kato, Nobuyoshi Azuma

**Affiliations:** 1 Department of Vascular Surgery, Asahikawa Medical University, Asahikawa, Hokkaido, Japan; 2 Department of Surgery, Pippu Clinic, Pippu town, Kamikawa-gun, Hokkaido, Japan

**Keywords:** vascular trauma, arteriovenous fistula, heart failure

## Abstract

Post-traumatic arteriovenous fistula (AVF) is a vascular injury complication and can present with vessel dilation, forming pulsatile varices, venous hypertension, distal ischemia, and congestive heart failure. We present a case of only pulsatile mass and leg pain caused by a 60-year-old post-traumatic AVF. Computed tomography angiography showed an AVF between the superficial femoral artery and superficial femoral vein. Surgical repair with AVF ligation was successfully performed. Traumatic AVF caused vascular and heart failure in the future; therefore, post-traumatic AVF is better eliminated as soon as possible.

## Introduction

Post-traumatic arteriovenous fistula (AVF) is a common complication of vascular trauma, especially low-energy trauma being the most important cause of traumatic AVF.^1,2)^ Recently, traumatic AVF has been reportedly symptomatic, and surgery was required several decades post-trauma. Furthermore, severe limb symptoms and heart failure have been reported.^3–5)^ Here, we describe a case of traumatic AVF with limb symptoms 60 years after a stabbing injury, which was successfully treated by surgical repair. Written informed consent was obtained from the patient for the publication of this case report and accompanying images.

## Case Report

**Figure 1:**
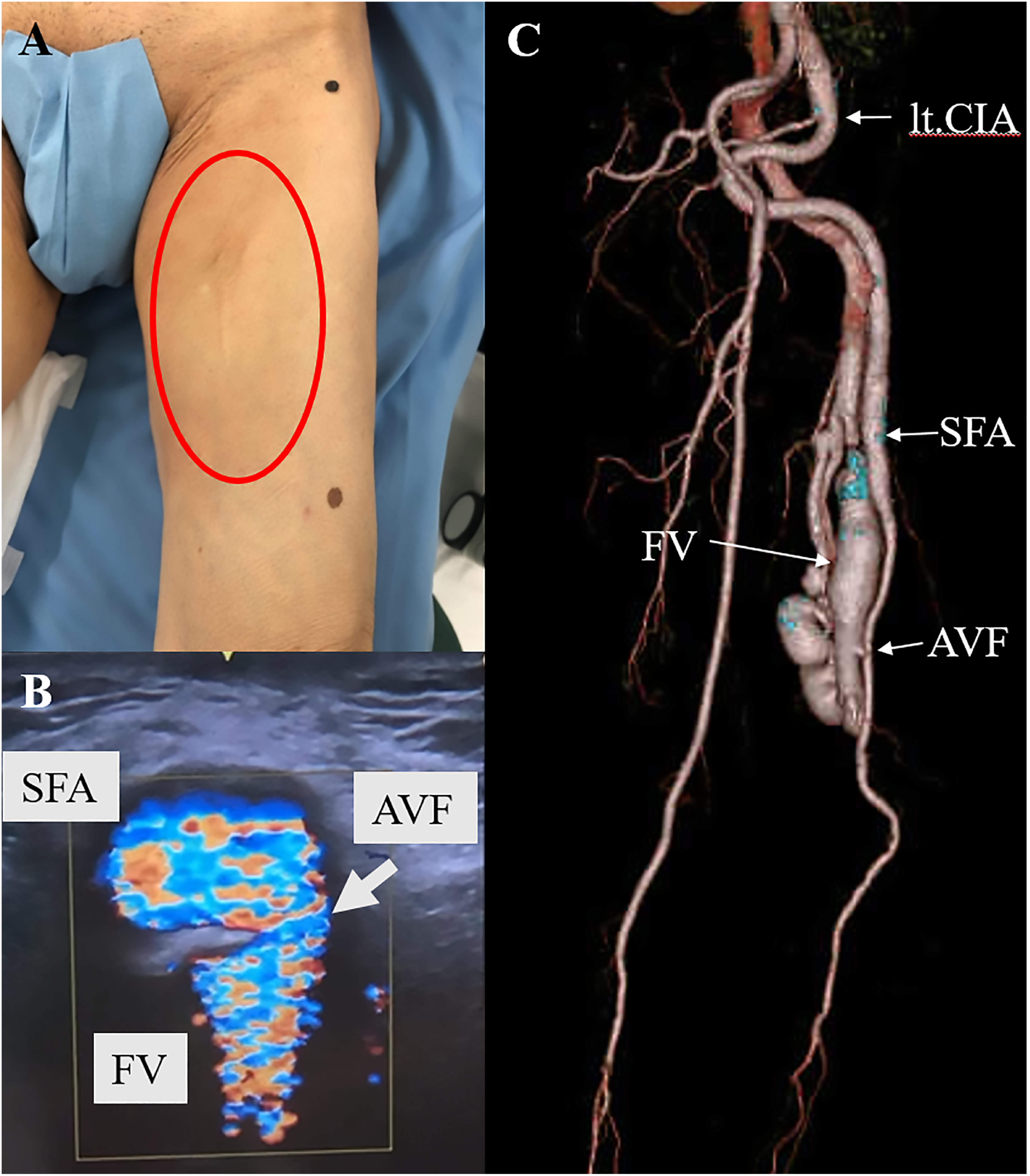
Fig. 1 Preoperative examination. (**A**) Preoperative condition of the upper extremity. (**B**) Echography shows the femoral arteriovenous fistula (AVF). (**C**) Preoperative computed tomography angiography shows AVF and dilation of the femoral vein (FV). Dilation was also observed from the common iliac artery (CIA) to the superficial femoral artery (SFA).

An 80-year-old man complained of pulsatile mass and left thigh pain after sustaining a stabbing injury in the left thigh 60 years ago and achieved surgical hemostasis (**Fig. 1A**). He had noticed a pulsatile mass in his left thigh about a year ago. Over the previous month, the pulsatile mass had grown and the pain had increased. He had a history of hypertension (HT), diabetes mellitus, dyslipidemia (DLp), and smoking. Duplex scan examination revealed AVF between the left superficial femoral artery (SFA) and superficial femoral vein (SFV) (**Fig. 1B**).

**Figure 2:**
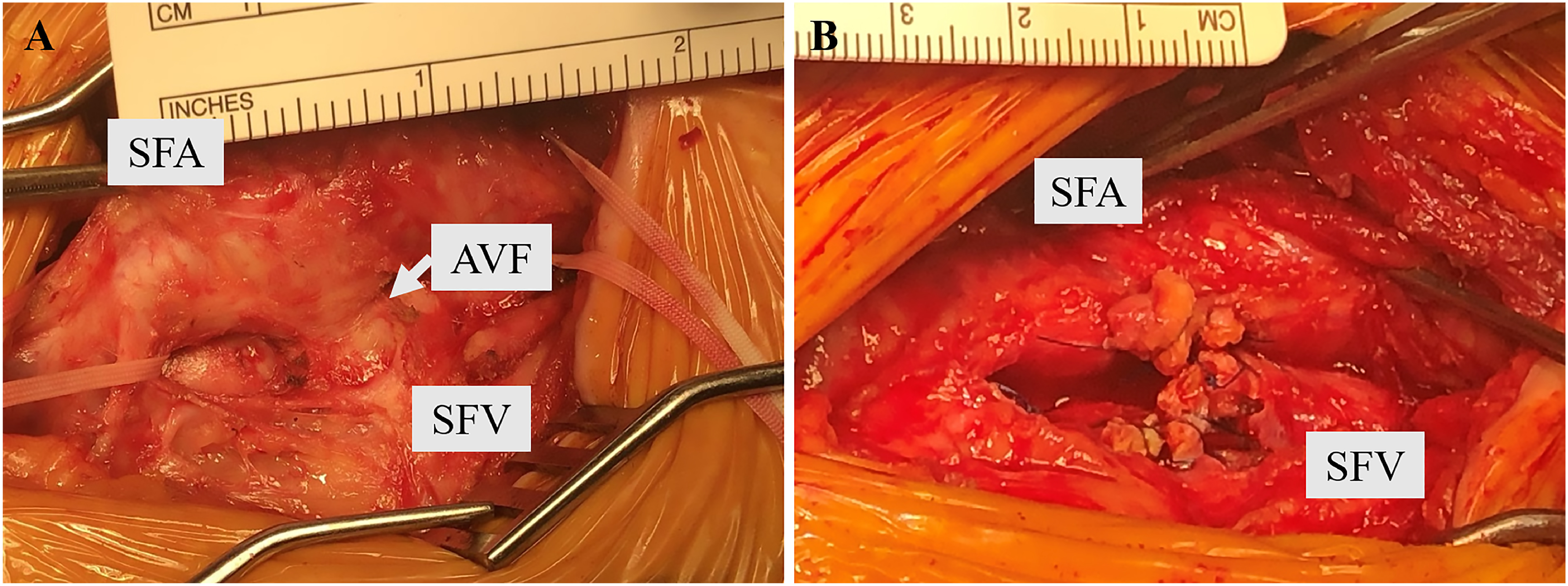
Fig. 2 Intraoperative view of the femoral arteriovenous fistula tract. (**A**) The fistula was found between the SFA and SFV. (**B**) The SFA and SFV were separated, and the fistula was closed.

SFA: superficial femoral artery; SFV: superficial femoral vein; AVF: arteriovenous fistula

**Figure 3:**
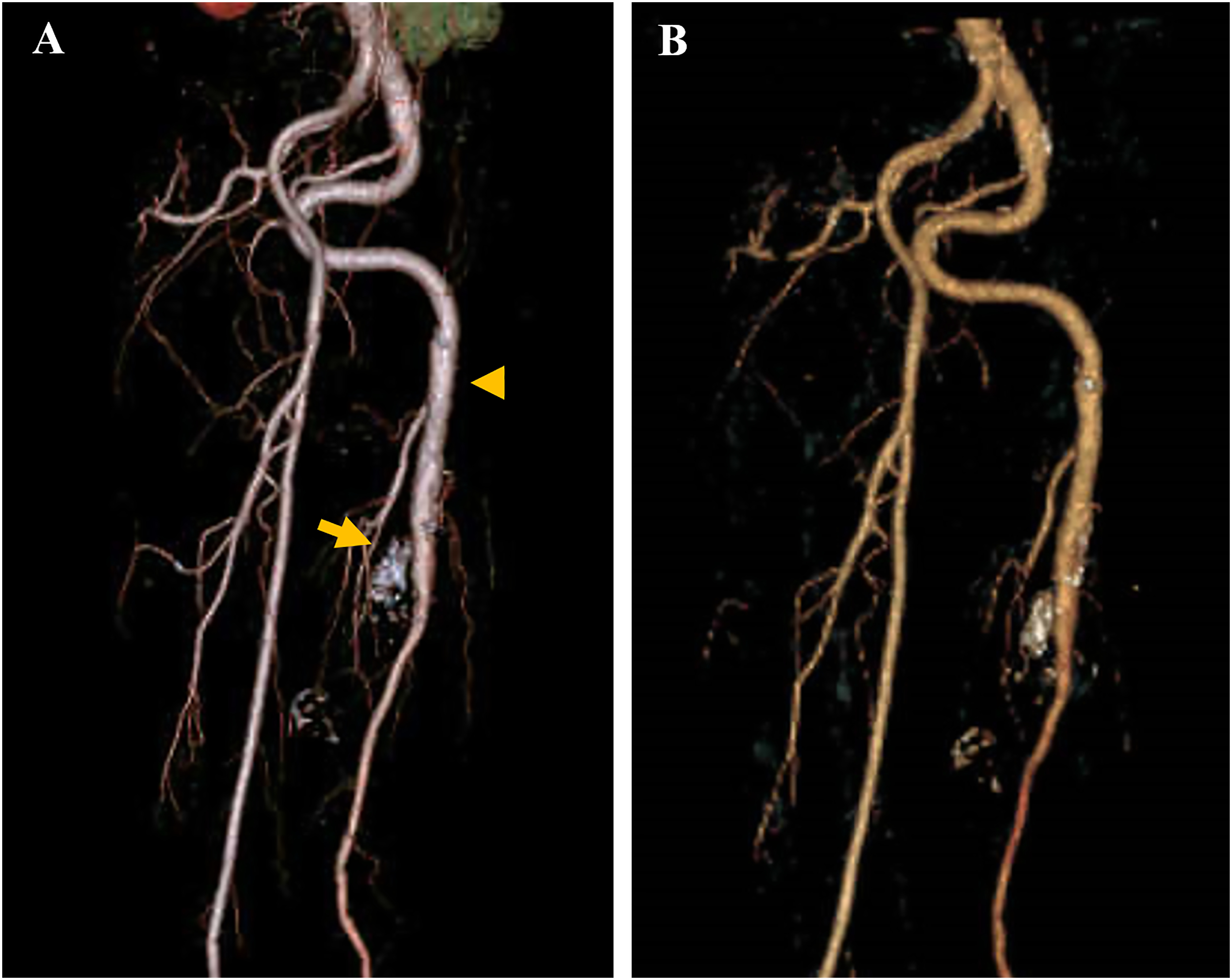
Fig. 3 Postoperative computed tomography angiography. (**A**) Postoperative image shows the absence of arteriovenous fistula (arrow), but notably persistent dilation of the left femoral artery (arrowhead). (**B**) Two years thereafter, the femoral artery did not change postoperatively.

Computed tomography (CT) revealed left lower extremity artery dilation from the common iliac artery to the SFA and femoral vein dilation through the AVF (**Fig. 1C**). His general condition was normal; however, the echocardiogram showed heart overload (increased left atrial dimension [LAD], 44 mm; ejection fraction [EF], 63%), the AVF was closed through open surgery. An incision was made on the medial side of the left thigh under general anesthesia. The fistula was found between the SFA and SFV (**Fig. 2A**). The proximal and distal ends of the vessels were clamped, the artery and vein were separated, and the fistula was closed using 4-0 and 5-0 polypropylene sutures (**Fig. 2B**). The postoperative course was uneventful, and the patient’s symptoms improved without the need for a postoperative antiplatelet or anticoagulant drug. CT angiography demonstrates an occluded AVF without flow. The common femoral artery and SFA dilatations (**Fig. 3A**) and dilated veins were also unchanged. Postoperatively, the D-dimer was 0.9 µg/mL; however, lower-leg edema did not occur. Thereafter, the patient was followed up for 2 years, and CT still showed a dilated artery (**Fig. 3B**). His left leg has never been edematous since the surgery, and ultrasonography showed no thrombosis in the dilated vein and negative D-dimer. Regarding the heart function, echocardiogram demonstrated an improved heart overload 2 years postoperatively, i.e., LAD of 37 mm and EF of 70%.

## Discussion

The incidence of AVF formation among all vascular injuries is 2.3%–3.5%. Trauma, especially low-energy trauma, such as stabbing and small-caliber gunshot injuries, is the most important cause of acquired AVF. Robbs et al. reported that stab wounds accounted for 63% of 202 traumatic AVFs. The most common anatomic sites of traumatic AVF are the neck and thoracic outlet (54%), upper limb (22%), and lower limbs (20%) arteries. Traumatic AVF usually presents with symptoms early after the trauma and requires treatment.^1,2)^ However, depending on the degree of trauma, AVF symptoms have been reported to appear years to decades post-injury. When the amount of the left and right shunt is high, vessel dilation is observed and caused limb symptoms. Iliac vein aneurysm with traumatic AVF and pseudoaneurysm also developed.^3,4)^ Furthermore, chronic volume overload to the heart causes remodeling, ventricular dilatation, and heart failure.^5)^ In this patient, heart overload due to traumatic AVF was asymptomatic, and AVF closure reduced the overload since LAD decreased after the surgery. However, a risk of future heart failure may exist due to cardiovascular risks. Furthermore, traumatic AVF could be the cause of ulcer formation in the lower limb.^6)^ Thus, early surgery could prevent the onset of heart failure and subsequent complications.

The treatment options for traumatic AVFs are surgical closure, stent graft, plugs and coil embolization, and echo-guided thrombin injection.^5–8)^ Some patients also reported hybrid treatment.^9)^ Although covered stent graft is anatomically a good indication for this patient, the postoperative dual-antiplatelet drug is required, which could be a disadvantage. The placement of coil embolization and plugs was difficult because of the short neck of the AVF. Finally, surgical repair was performed; however, if anatomically possible, these treatments can be performed in patients contraindicated for open surgery. Although traumatic AVF closure was performed, arteriomegaly may develop postoperatively in the artery, which dilated once through the AVF.^10)^ Consistent with our patient, careful follow-up is necessary for CT in the future because arterial dilation has not improved.

## Conclusion

We have reported a case of traumatic AVF occurring after 60 years of stabbing injury. Traumatic AVF may appear decades post-injury, could be the cause of several complications, and is better treated as soon as discovered.
